# Variations in the use of oxytocin for augmentation of labour in Sweden: a population-based cohort study

**DOI:** 10.1038/s41598-024-68517-1

**Published:** 2024-07-30

**Authors:** Karin Johnson, Kari Johansson, Charlotte Elvander, Sissel Saltvedt, Malin Edqvist

**Affiliations:** 1 Clinical Epidemiology Division, Department of Medicine Solna, Karolinska Institutet, Stockholm, Sweden; 2 Department of Women’s Health and Health Professions, Karolinska University Hospital, Stockholm, Sweden; 3 Department of Women’s and Children’s Health, Department of Medicine Solna, Karolinska Institutet, Stockholm, Sweden

**Keywords:** Robson classification, Medical interventions, Oxytocin augmentation, Epidural analgesia, Cohort study, Health care, Medical research

## Abstract

National Swedish data shows substantial variation in the use of oxytocin for augmentation of spontaneous labour between obstetric units. This study aimed to investigate if variations in the use of oxytocin augmentation are associated with maternal and infant characteristics or clinical factors. We used a cohort design including women allocated to Robson group 1 (nulliparous women, gestational week ≥ 37 + 0, with singleton births in cephalic presentation and spontaneous onset of labour) and 3 (parous women, gestational week ≥ 37 + 0, with singleton births in cephalic presentation, spontaneous onset of labour, and no previous caesarean birth). Crude and adjusted logistic regression models with marginal standardisation were used to estimate risk ratios (RR) and risk differences (RD) with 95% confidence intervals (CI) for oxytocin use by obstetric unit. An interaction analysis was performed to investigate the potential modifying effect of epidural. The use of oxytocin varied between 47 and 73% in Robson group 1, and 10% and 33% in Robson group 3. Compared to the remainder of Sweden, the risk of oxytocin augmentation ranged from 13% lower (RD − 13.0, 95% CI − 15.5 to − 10.6) to 14% higher (RD 14.0, 95% CI 12.3–15.8) in Robson group 1, and from 6% lower (RD − 5.6, 95% CI − 6.8 to − 4.5) to 18% higher (RD 17.9, 95% CI 16.5–19.4) in Robson group 3. The most notable differences in risk estimates were observed among women in Robson group 3 with epidural. In conclusion, variations in oxytocin use remained despite adjusting for risk factors. This indicates unjustified differences in use of oxytocin in clinical practice.

## Introduction

Oxytocin is a reproductive hormone involved in the process of childbirth. Its synthetic equivalent is widely used to induce or augment labour, aiming to increase the frequency, duration, and strength of uterine contractions, thereby accelerating the birth progress^1,2^. It represents the sole medical intervention available for treating labour dystocia and has been shown to reduce the mean duration of labour in several clinical trials^2–4^. However, there is a lack of evidence supporting its efficacy in reducing caesarean or instrumental births^2,5^. When stratified by maternal age, the use of oxytocin has been associated with a reduced risk of caesarean births among women with epidural analgesia in Norway and Denmark^6^. Epidural analgesia is closely linked to oxytocin augmentation as women who receive epidurals more often experience longer labours, thus increasing the likelihood of labour augmentation^7,8^. Further risk factors associated with the need for oxytocin augmentation in spontaneous onset of labour include primiparity, gestational age ≥ 42 weeks, infant birthweight ≥ 4000 g, advanced maternal age, obesity, and foetal malpresentation^9,10^.

Synthetic oxytocin increases the risk of harm to infants and women^11^, with adverse side effects including neonatal acidosis, asphyxia, and uterine rupture^12,13^. The clinical challenge posed by oxytocin administration stems from the drug's broad individual therapeutic range, where the same dosage may yield no effect in some women while causing hypertonic uterine activity in others^1^. Intrapartum use of oxytocin has been reported to be frequently misapplied, with instances of excessive dosing and untimely administration^14^. Inappropriate oxytocin administration in obstetrics has been associated with adverse infant outcomes, attributable to both malpractice and challenges in interpreting foetal well-being. Due to the risk profile, oxytocin has been classified as a high-risk medication since 2007^15^.

National public data from the Swedish Pregnancy Register in 2022 show considerable disparities in the use of oxytocin across obstetric units. Among primiparous and multiparous women with spontaneous start of labour, the use of oxytocin varied from 43 to 65% and from 16 to 37%, respectively^16^. Considering the potency of oxytocin and its association with adverse maternal and infant outcomes, we wanted to investigate whether known risk factors for oxytocin use are the primary drivers of the observed national variations, or if other factors are involved. Therefore, this study aimed to investigate whether the variations between obstetric units in use of oxytocin for augmenting spontaneous labour are associated with maternal and infant characteristics or clinical factors.

## Methods

### Study design and population

This is a nationwide cohort study using data from the Swedish Pregnancy Register, a national quality register that includes information on 98.4% of all births in Sweden. Maternal and infant information including data on maternal demographics, pregnancy and labour characteristics, and infant outcomes is prospectively collected^17^. In Sweden, maternity care is funded by taxes and provided free of charge (Table S5). The majority of women give birth in hospitals, in obstetric units that receive public funding. The smallest obstetric units only provide care for women with low risk and will refer women with high risk to an obstetric unit located at a university teaching hospital. Midwives are the primary caregivers regardless of risk, and for women considered intermediate or high-risk, they work in close collaboration with obstetricians. All obstetric units follow a risk classification system^18^ and in cases of labour dystocia, where oxytocin is prescribed, the woman is considered at higher risk and will therefore be monitored with continuous CTG. While all obstetric units have guidelines for managing labour dystocia and the use of oxytocin, national guidelines are currently lacking.

For the present study, the population comprised women who gave birth between January 1, 2018, and December 31, 2021. The Robson classification^19^ was employed to define the two subgroups of interest: nulliparous women at term (≥ 37 + 0 weeks), with singleton births in cephalic presentation and spontaneous onset of labour (Robson group 1), and parous women at term (≥ 37 + 0 weeks), with singleton births in cephalic presentation, spontaneous onset of labour, and no previous caesarean birth (Robson group 3). Women giving birth at obstetric units with incomplete data transfer regarding oxytocin augmentation during the specified time frame (n = 4), and women with intrauterine foetal demise were excluded (Fig. S1).

There was no user involvement from women in the design, interpretation or reporting of this study. The study was approved by the Regional Ethics Committee in Stockholm (2020-02500 and 2021-06055-02).

### Analysis of oxytocin use for augmentation of labour by obstetric unit

The outcome of interest, i.e., the use of oxytocin for augmentation of spontaneous labour, was identified using the Swedish version of the ICD-10 code DT037, and the variable “Oxytocin during labour” (yes/no), both variables available in the Swedish Pregnancy Register. The independent variable was the obstetric unit, defined as all obstetric units for which the data reporting on oxytocin augmentation to the Swedish Pregnancy Register for the study period was reliable (n = 40).

### Covariates

#### Confounders

Covariates related to the woman encompassed age, early pregnancy body mass index (BMI), height, education level, and country of birth. Covariates related to the infant included birthweight and gestational week at birth. To investigate whether the obstetric unit's size influenced oxytocin use, the variable “annual birth rate” was used, where a lower annual birth rate corresponds to a smaller obstetric unit. This variable was categorised into < 1000, 1000–2499, 2500–3999, and ≥ 4000 births per year. Other continuous variables categorised were: maternal age (< 25, 25–29, 30–34, ≥ 35), maternal height (< 155, 155–164, 165–174, ≥ 175), education level (no formal schooling, primary, secondary, post-secondary), country of birth (Nordic, Asia, Africa, South America, other), infant birthweight (< 3000, 3000–3499, 3500–3999, ≥ 4000), and gestational week (37 + 0–38 + 6, 39 + 0–40 + 6, 41 + 0–41 + 6, ≥ 42). BMI was calculated from weight and height squared at the first prenatal visit and categorised (underweight < 18.5, normal weight 18.5–24.9, overweight 25–29.9, and obese ≥ 30).

#### Effect modifier

Due to its close relationship with oxytocin, epidural analgesia was considered an effect modifier. Information regarding the use of epidural analgesia (yes/no) was derived from the ICD-10 code SN999.

### Statistical analysis

Descriptive statistics with means, standard deviations (SD), or percentages were used to present the use of oxytocin by maternal and infant characteristics, as well as the hospital's annual birth rate. All analyses were stratified according to Robson groups 1 and 3. To explore the relationship between previously identified factors related to labour augmentation, as well as the variations in its usage, crude and adjusted risk ratios (RR) and the 95% confidence interval (95% CI) were estimated using logistic regression with marginal standardisation^20^. The variables considered for the regression model included maternal age, BMI, height, education level, country of birth, birthweight, gestational week, and birth rate. This model was subsequently employed to estimate crude and adjusted risk ratios for the use of oxytocin at each obstetric unit in comparison to the remainder of Sweden. Additionally, to further elucidate the difference in the percent of augmentation across obstetric units in comparison with the rest of Sweden, both crude and adjusted risk differences (RD) with 95% CIs were calculated using the same model (Tables S2 and S3).

To investigate the potential modifying effect of epidural anaesthesia on the use of oxytocin augmentation, an interaction analysis was conducted. In the presence of a significant interaction effect, the relationship between the obstetric unit and the use of oxytocin was examined separately among women who received epidural analgesia and those who did not. The same adjustments applied in the previous model were adapted. Stata version 17.0 was used for all statistical analyses. All methods were performed in accordance with the relevant guidelines and regulations.

### Ethics approval

The study was approved by the Regional Ethical Committee (IRB) at Karolinska Institutet, Stockholm (2020-02500 and 2021-06055-02). It is based on register data where serial numbers have replaced personal identification numbers. All data management and analyses were conducted on de-identified data. No informed consent was needed.

## Results

### Study population

The original cohort included 256,323 women. After excluding 14,532 women who gave birth in an obstetric unit with uncertain reporting of data on oxytocin, and 183 women with intrauterine foetal demise, the final study population included 241,608 women. Among these, 106,676 (44%) were in Robson group 1, and 134,932 (56%) in Robson group 3 (Fig. S1).

The rate of oxytocin for augmentation of labour was 59% in Robson group 1 and 16% in Robson group 3 (Table 1). In both Robson groups, maternal and infant characteristics were associated with the use of oxytocin augmentation, as it increased among older women (≥ 35 years of age), women who were obese (BMI ≥ 30), shorter women (≤ 155 cm), women of non-Nordic origin, and among women who gave birth to an infant of higher birthweight (≥ 4000 g) (Table 1). Furthermore, oxytocin was used to a higher extent in obstetric units with an annual birth rate of less than 1000 births (Table 1). Additionally, epidural analgesia significantly increased the risk of oxytocin use, with a doubled risk in Robson group 1 and a threefold risk in Robson group 3 (Table S1).

**Table 1:** Risk factors for oxytocin augmentation during labour among 241 608 women in Robson groups 1 and 3, Sweden, 2018–2021.

	Robson group 1 n (%) = 106 676 (44.1)		Robson group 3 n (%) = 134 932 (55.9)	
	Oxytocin augmentation n (%) = 63 283 (59.3)	No oxytocin augmentation n (%) = 43 393 (40.7)	Oxytocin augmentation n (%) = 21 213 (15.7)	No oxytocin augmentation n (%) = 113 719 (84.3)
	n (row %)	n (row %)	n (row%)	n (row %)
Maternal characteristics				
Age (years)				
< 25	8891 (50.3)	8845 (49.7)	1207 (14.9)	6908 (85.1)
25–29	24,976 (58.7)	17,578 (41.3)	5599 (15.1)	31,395 (84.9)
30–34	21,707 (62.5)	13,018 (37.5)	8249 (15.1)	46,397 (84.9)
≥ 35	7655 (66.0)	3942 (34.0)	6155 (17.5)	29,008 (82.5)
BMI (kg/m^2^)				
Underweight (< 18.5)	1666 (53.7)	1438 (46.3)	388 (12.2)	2794 (87.8)
Normal weight (18.5–24.9)	37,096 (58.9)	25,851 (41.1)	10,658 (14.6)	62,242 (85.4)
Overweight (25–29.9)	14,882 (61.1)	9466 (38.9)	5862 (16.8)	29,138 (83.3)
Obese (≥ 30)	6429 (61.8)	3974 (38.2)	3481 (19.3)	14,553 (80.7)
Missing	3210 (5.1)	2664 (6.1)	824 (3.9)	4992 (4.4)
Height (cm)				
< 155	2280 (67.2)	1112 (32.8)	1168 (23.6)	3774 (76.4)
155–164	22,584 (62.0)	13,839 (38.0)	8753 (17.8)	40,529 (82.2)
165–174	30,207 (58.5)	21,410 (41.5)	9248 (14.3)	55,442 (85.7)
≥ 175	6043 (54.3)	5095 (45.7)	1542 (12.5)	10,840 (87.6)
Missing	2169 (3.4)	1937 (4.5)	502 (2.4)	3134 (2.8)
Education level				
No formal schooling	446 (53.0)	395 (47.0)	661 (19.7)	2694 (80.3)
Primary	2146 (55.1)	1749 (44.9)	1378 (18.6)	6047 (81.4)
Secondary	18,493 (58.3)	13,221 (41.7)	6914 (16.6)	34,818 (83.4)
Post-secondary	32,984 (61.0)	21,181 (39.1)	8779 (14.4)	52,307 (85.6)
Missing	9214 (14.6)	6847 (15.8)	3481 (16.4)	17,853 (15.7)
Country of birth				
Nordic	44,238 (58.8)	30,972 (41.2)	12,674 (14.6)	73,914 (85.4)
Asia	2026 (65.1)	1087 (34.9)	767 (18.2)	3442 (81.8)
Africa	1747 (57.8)	1274 (42.2)	1748 (20.4)	6805 (79.6)
South America	482 (69.0)	217 (31.0)	192 (22.4)	666 (77.6)
Other	9133 (61.2)	5793 (38.8)	3920 (17.0)	19,136 (83.0)
Missing	5657 (8.9)	4050 (9.3)	1912 (9.0)	9756 (8.6)
Epidural analgesia				
Yes	49,496 (76.3)	15,338 (23.7)	9798 (35.0)	18,176 (65.0)
Infant characteristics				
Birthweight (g)				
< 3000	6149 (46.2)	7169 (53.8)	1163 (11.8)	8720 (88.2)
3000–3499	23,510 (55.8)	18,607 (44.2)	5603 (13.1)	37,141 (86.9)
3500–3999	24,062 (63.7)	13,712 (36.3)	8623 (15.9)	45,563 (84.1)
≥ 4000	9229 (71.6)	3661 (28.4)	5737 (20.9)	21,695 (79.1)
Missing	333 (0.5)	244 (0.6)	87 (0.4)	600 (0.5)
Gestational age at birth (weeks)				
37 + 0–38 + 6	8431 (48.1)	9091 (51.9)	3014 (13.3)	19,733 (86.8)
39 + 0–40 + 6	39,347 (58.8)	27,563 (41.2)	13,490 (15.1)	75,707 (84.9)
41 + 0–41 + 6	14,263 (69.2)	6364 (30.8)	4340 (19.9)	17,441 (80.1)
≥ 42	1242 (76.8)	375 (23.2)	369 (30.6)	838 (69.4)
Hospital annual birth rate				
< 1000	4029 (61.7)	2505 (38.3)	1831 (18.8)	7924 (81.2)
1000–2499	19,367 (59.2)	13,363 (40.8)	7360 (16.6)	36,871 (83.4)
2500–3999	21,399 (60.2)	14,139 (39.8)	7311 (16.3)	37,467 (83.7)
≥ 4000	18,488 (58.0)	13,386 (42.0)	4711 (13.0)	31,457 (87.0)

### Variations in oxytocin use between obstetric units

**Figure 1:**
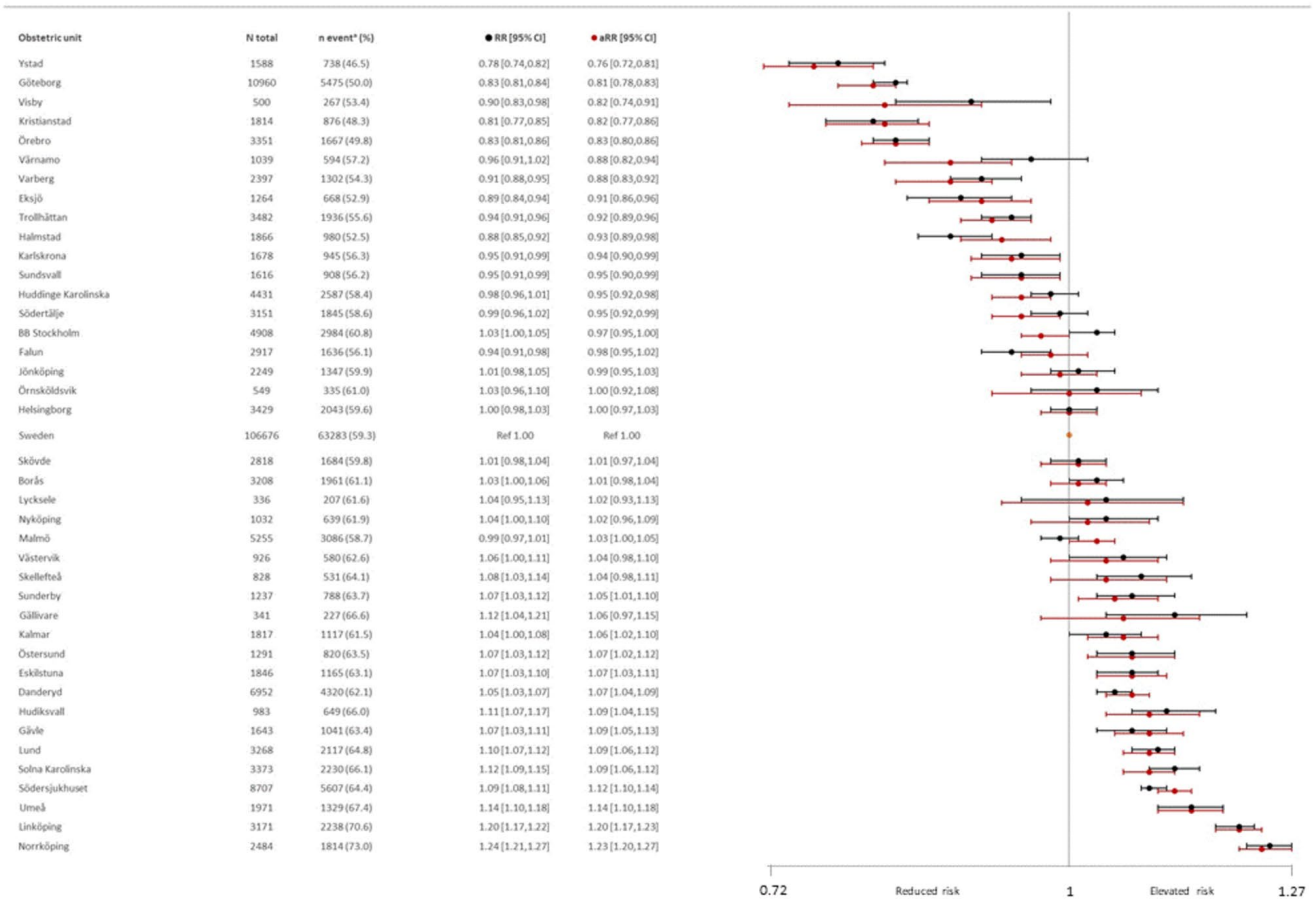
Crude and adjusted risk ratios and 95% confidence intervals for oxytocin augmentation during labour by 40 obstetric units among 106,676 women in Robson group 1, Sweden, 2018–2021. Adjusted risk ratios (aRR), in red, were adjusted for maternal age, BMI, height, education level, country of birth, infant birthweight, gestational week, and hospital annual birth rate. The reference value is the rest of Sweden, indicated as the grey line.

In Robson group 1, the use of oxytocin varied between 47 and 73% among the 40 obstetric units, with an overall prevalence of 59% (Fig. 1, Table S2). In the obstetric unit with the lowest use, the absolute risk of oxytocin augmentation was 13% lower than in the rest of Sweden (RD − 13.04, 95% CI − 15.51 to − 10.57). In the obstetric unit with the highest use, the absolute risk of oxytocin was 14% higher (RD 14.03, 95% CI 12.26–15.80). In 11 of the units, there was no statistically significant difference in oxytocin use as compared to the rest of Sweden (Table S2). Adjusting for maternal and infant characteristics as well as the annual birth rate did not change these associations, indicating that variations in oxytocin use were driven by other factors. For example, the crude and adjusted estimates for the unit with the lowest (738/1588; 47%), as well as the highest (1814/2484; 73.0%) use of oxytocin, were almost identical (RR 0.78, 95% CI 0.74–0.82; aRR 0.76, 95% CI 0.72–0.81) and (RR 1.24, 95% CI 1.21–1.27; aRR 1.23, 95% CI 1.20–1.27) (Fig. 1).

**Figure 2:**
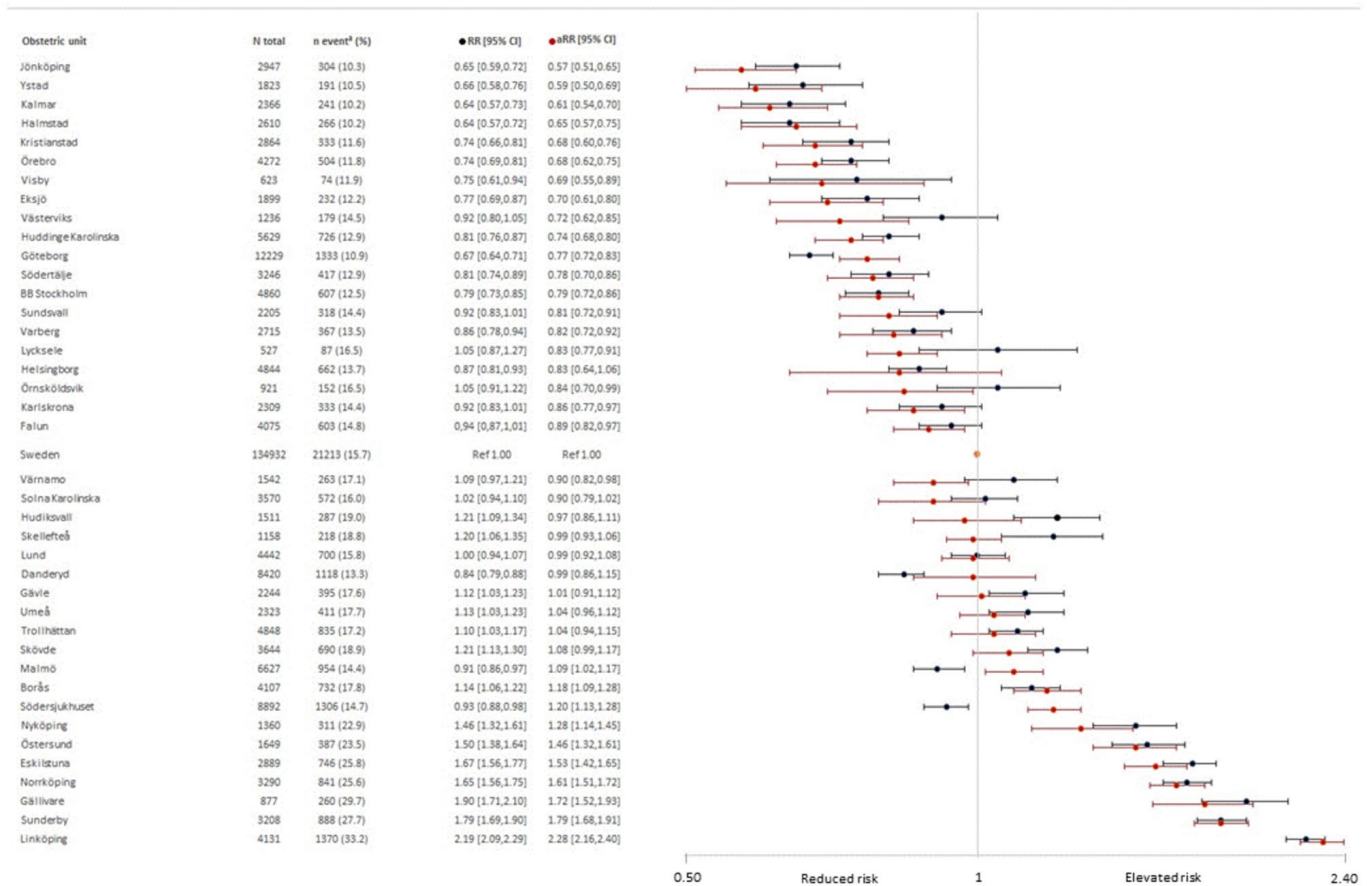
Crude and adjusted risk ratios and 95% confidence intervals for oxytocin augmentation during labour by 40 obstetric units among 134,932 women in Robson group 3. Sweden, 2018–2021. Adjusted risk ratios (aRR), in red, were adjusted for maternal age, BMI, height, education level, country of birth, infant birthweight, gestational week, and hospital annual birth rate. The reference value is the rest of Sweden, indicated as the grey line.

In Robson group 3, the use of oxytocin varied between 10 and 33% among the 40 units, with an overall prevalence of 16% (Fig. 2, Table S3). In this group, the absolute risk of oxytocin augmentation in the obstetric unit with the lowest use was 6% lower as compared to the rest of Sweden (RD − 5.64, 95% CI − 6.82 to − 4.46). In the obstetric unit with the highest use, the absolute risk of oxytocin augmentation was 18% higher (RD 17.99, 95% CI 16.54–19.44). In nine of the units, there was no statistically significant difference in oxytocin use as compared to the rest of Sweden (Table S3). For women in Robson group 3, adjustments resulted in a broader range of risk estimates compared to Robson group 1 and increased the variation among the obstetric units. In six of the units, the risk estimates shifted from an elevated risk to indicating a reduced risk as compared to the remainder of Sweden. However, in each case, there was an overlap in CIs, indicating that the observed associations lacked statistical significance. Conversely, for two of the units, the risk estimates changed from displaying a reduced risk to showing an elevated risk, and this alteration in risk was driven by hospital size, i.e., annual birth rate (Fig. 2).

### Use of oxytocin augmentation in relation to the use of epidural analgesia

The results of the interaction analysis indicated a significant interaction effect between the use of epidural analgesia and oxytocin augmentation in 16 out of 40 units in Robson group 1 and 22 out of 40 units in Robson group 3 (Table S4). In Robson group 1, the utilisation of oxytocin in Sweden overall differed notably between women without epidural (22%) and those with epidural (78%). Conversely, in Robson group 3, the usage of oxytocin was more comparable between women who did not receive epidurals (54%) and those who did (46%) (Table 2).

**Table 2:** Adjusted risk differences and 95% confidence intervals for oxytocin augmentation during labour by use of epidural analgesia among obstetric units. Women in Robson groups 1 and 3, Sweden, 2018–2021.

Obstetric unit	Oxytocin augmentation Robson group 1					*p* value interaction^d^
	N total	No epidural analgesia		Epidural analgesia		
		n (%) event^a^	Adjusted risk difference^b^ (95% CI)	n (%) event^c^	Adjusted risk difference^b^ (95% CI)	
Sweden	63,283	13,787 (22)	1.00 (ref)	49,496 (78)	1.00 (ref)	1.00 (ref)
Örebro	1667	425 (25)	− 1.49 (− 4.26 to 1.28)	1242 (75)	− 14.93 (− 17.24 to − 12.61)	< 0.001
Värnamo	594	164 (28)	− 2.63 (− 7.57 to 2.32)	430 (72)	− 10.16 (− 14.69 to − 5.62)	< 0.001
Sundsvall	908	212 (23)	0.69 (− 3.44 to 4.82)	696 (77)	− 8.64 (− 11.77 to − 5.52)	< 0.001
Eksjö	668	279 (42)	4.99 (1.10–8.88)	389 (58)	− 4.19 (− 8.23 to − 0.15)	< 0.001
Trollhättan	1936	501 (26)	0.12 (− 2.70 to 2.94)	1435 (74)	− 4.02 (− 6.24 to − 1.79)	0.001
BB Stockholm	2984	343 (11)	− 13.76 (− 15.96 to − 11.55)	2641 (89)	− 0.45 (− 2.15 to 1.26)	< 0.001
Huddinge Karolinska	2587	550 (21)	− 3.77 (− 6.17 to − 1.36)	2037 (79)	0.00 (− 1.88 to 1.88)	0.036
Eskilstuna	1165	358 (31)	9.51 (5.78–13.23)	807 (69)	1.39 (− 1.39 to 4.18)	< 0.001
Danderyd	4320	520 (12)	− 4.77 (− 7.21 to − 2.33)	3800 (88)	1.85 (0.40–3.29)	< 0.001
Södersjukhuset	5607	689 (12)	− 0.84 (− 3.16 to 1.48)	4918 (88)	3.01 (1.70–4.31)	< 0.001
Borås	1961	441 (22)	0.02 (− 2.91 to 2.96)	1520 (78)	4.05 (2.01–6.09)	0.062
Solna Karolinska	2230	255 (11)	− 8.38 (− 11.48 to − 5.29)	1975 (89)	4.30 (2.42–6.18)	< 0.001
Linköping	2238	410 (18)	13.70 (10.09–17.31)	1828 (82)	4.72 (2.95–6.50)	< 0.001
Norrköping	1814	342 (19)	15.03 (10.96–19.11)	1472 (81)	5.35 (3.39–7.31)	< 0.001
Malmö	3086	971 (31)	12.73 (10.24–15.21)	2115 (69)	7.26 (5.60–8.92)	0.025
Gällivare	227	54 (24)	− 3.58 (− 11.14 to 3.99)	173 (76)	9.94 (4.86–15.02)	0.041

Obstetric unit	Oxytocin augmentation Robson group 3					*p* value interaction^d^
	N total	No epidural analgesia		Epidural analgesia		
		n (%) event^a^	Adjusted risk difference^b^ (95% CI)	n (%) event^c^	Adjusted risk difference^b^ (95% CI)	
Sweden	21,213	11,415 (54)	1.00 (ref)	9798 (46)	Ref 1.00	1.00 (ref)
Jönköping	304	125 (41)	− 6.14 (− 7.06 to − 5.23)	179 (59)	− 8.83 (− 12.63 to − 5.03)	< 0.001
BB Stockholm	607	156 (26)	− 5.82 (− 6.65 to − 4.98)	451 (74)	− 7.57 (− 10.14 to − 4.99)	0.002
Solna Karolinska	572	158 (28)	− 4.99 (− 5.98 to − 4.00)	414 (72)	− 4.80 (− 7.84 to − 1.76)	0.001
Huddinge Karolinska	726	317 (44)	− 4.69 (− 5.46 to − 3.92)	409 (56)	− 4.57 (− 7.43 to − 1.71)	0.003
Ystad	191	107 (56)	− 4.49 (− 5.78 to − 3.20)	84 (44)	− 4.55 (− 10.96 to 1.86)	0.037
Danderyd	1118	382 (34)	− 1.80 (− 2.84 to − 0.75)	736 (66)	− 3.54 (− 5.96 to − 1.13)	0.001
Nyköping	311	224 (72)	5.82 (3.38 to 8.25)	87 (28)	− 3.33 (− 9.77 to 3.11)	< 0.001
Östersund	387	216 (56)	6.73 (4.38–9.09)	171 (44)	− 1.95 (− 6.41 to 2.52)	< 0.001
Göteborg	1333	645 (48)	− 2.15 (− 3.06 to − 1.24)	688 (52)	− 1.17 (− 3.87 to 1.53)	< 0.001
Södersjukhuset	1306	435 (33)	0.23 (− 0.94 to 1.39)	871 (67)	− 0.59 (− 2.97 to 1.78)	0.036
Gävle	395	161 (41)	− 2.78 (− 4.14 to − 1.41)	234 (59)	0.08 (− 4.06 to 4.23)	0.003
Trollhättan	835	606 (73)	2.79 (1.59–3.98)	229 (27)	0.74 (− 3.58 to 5.05)	< 0.001
Karlskrona	333	162 (49)	− 3.45 (− 4.78 to − 2.11)	171 (51)	0.91 (− 3.86 to 5.68)	0.001
Norrköping	841	479 (57)	8.73 (7.01–10.45)	362 (43)	3.11 (− 0.36 to 6.58)	< 0.001
Umeå	411	180 (44)	− 1.71 (− 3.14 to − 0.27)	231 (56)	4.07 (− 0.39 to 8.53)	0.002
Eskilstuna	746	536 (72)	9.47 (7.74–11.20)	210 (28)	5.15 (0.29–10.02)	< 0.001
Halmstad	266	196 (74)	− 2.94 (− 4.23 to − 1.64)	70 (26)	9.25 (0.32–18.17)	< 0.001
Malmö	954	602 (63)	4.82 (3.38–6.26)	352 (37)	12.20 (8.25–16.15)	0.001
Linköping	1370	851 (62)	18.46 (16.56–20.36)	519 (38)	14.55 (11.20–17.89)	< 0.001
Borås	732	456 (62)	2.62 (1.30–3.93)	276 (38)	15.59 (10.84–20.34)	0.048
Sunderby	888	404 (45)	6.56 (4.88–8.24)	484 (55)	19.39 (15.70–23.08)	0.017
Gällivare	260	130 (50)	4.91 (2.13–7.68)	130 (50)	30.73 (23.53–37.92)	0.001

Adjusted risk difference contrasted the risk of oxytocin augmentation at each obstetric unit versus the rest of Sweden.

^a^Number of women without epidural analgesia and oxytocin augmentation.

^b^Adjusted for maternal age, BMI, height, education level, country of birth, infant birthweight, gestational week, and hospital annual birth rate.

^c^Number of women with epidural analgesia and oxytocin augmentation.

^d^Interaction effect between obstetric unit and epidural analgesia on oxytocin augmentation during labour (Table S4).

Within Robson group 1, the absolute risk of oxytocin augmentation remained relatively consistent regardless of epidural use. For women without epidural, the spread in risk ranged from 14% lower to 15% higher as compared to the rest of Sweden (aRD − 13.76, 95% CI − 15.96 to − 11.55; aRD 15.03, 95% CI 10.96–19.11) (Table 2). In this group, 7 out of the 16 units showed no statistically significant differences in oxytocin use as compared to the remainder of Sweden, while the usage was lower in 4 units and higher in 5 units (Table 2).

In contrast, among women in Robson group 3, the variation in risk of oxytocin use between obstetric units was more pronounced for those with epidural, with differences in risk ranging from 9% lower to 31% higher compared to the remainder of Sweden (aRD − 8.83, 95% CI − 12.63 to − 5.03; aRD 30.73, 95% CI 23.53–37.92) (Table 2). Out of the 22 units where an interaction effect between the obstetric unit and use of epidural was observed, the stratified analysis showed no statistically significant differences in oxytocin use for women with epidural in 10 of the units, while oxytocin use was lower in 5 units and higher in 7 units compared to the rest of Sweden (Table 2).

## Discussion

### Interpretation

This study showed that among women with spontaneous onset of labour, variations in the use of oxytocin augmentation across the obstetric units persisted despite adjusting for maternal and infant characteristics and the hospital annual birth rate. Overall, variations were more pronounced in Robson group 3 than in Robson group 1, with the most substantial differences in risk of oxytocin augmentation observed among parous women with epidural analgesia.

Even after adjusting for factors known to increase the risk of augmentation, our results revealed that variations in the use of oxytocin for augmenting spontaneous labour persisted among obstetric units. This finding is in line with previous research, indicating that the use of medical interventions during labour and birth varies between countries as well as among regions and individual providers^21–23^. Our results, however, differ from those of Seijmonsbergen et al., who found that variations in oxytocin use between high-income countries were attributed to differences in BMI and ethnicity^21^. In contrast to our study, they focused on comparing medical interventions between countries rather than between obstetric units within the same country. Even though characteristics of childbearing women such as BMI, age, and education level are known to vary between rural and urban areas in Sweden^23^, variation is likely less pronounced when comparing rates of interventions within the same country. Our results further align with those of Mesterton and colleagues, which showed persisting inter-hospital variations in labour and birth outcomes after adjusting for case-mix^24^.

Compared to women giving birth in obstetric units with 1000–2499 births per year, the risk of oxytocin augmentation was slightly increased among women who gave birth in units with less than 1000 births. When adjusting for risk factors related to oxytocin, no significant change in risk occurred until we adjusted for annual birth rate. Our hypothesis posited that women giving birth in units with a lower annual birth rate would have the lowest risk of being augmented with oxytocin. This assumption was based on the premise that these women would experience less stress from caregivers as larger units are known to be associated with increased stress among clinicians^25^, potentially influencing their decisions to intervene. Conversely, it was anticipated that obstetricians and midwives working in smaller units would be less inclined to intervene, as these units often have a specific commission for women considered low-risk.

Although the results for women in Robson groups 1 and 3 showed a similar pattern regarding risk of oxytocin augmentation, the variation in use was more pronounced in Robson group 3. This is noteworthy, as both epidural use and labour dystocia are more prevalent in nulliparous women^10,21,26^ suggesting that the reverse pattern should have been observed. Furthermore, the interaction analysis, which was significant for the majority of the obstetric units, revealed substantial variation in risk estimates among women in Robson group 3 with epidural. Midwives report that augmentation of oxytocin is more likely to occur in instances of high workload or the absence of guidelines or protocols^27^. While our study lacks data on high workload, it is hard to assume that the impact of this factor would be limited solely to oxytocin use within this particular group of women. As there would be no physiological rationale for women in Robson group 3 to require oxytocin to a greater extent than women in Robson group 1^10^, our findings suggest that additional factors influence the decision-making process. Previous suggestions for explanatory factors comprise both organisational and/or other non-clinical factors, including the culture of care^28,29^.

There are no national guidelines for labour dystocia in Sweden, and this might potentially be reflected in our results, as local policies affect clinicians’ decisions^24^. In addition, individual clinicians’ knowledge and experience, as well as opinions from senior colleagues’ are known to impact decision-making regarding labour augmentation^30,31^. Previous studies have demonstrated variations in the diagnosis and interpretation of labour dystocia with clinicians using oxytocin even in cases that, by definition, did not involve labour arrest^14,32,33^. Qualitative data from Sweden further revealed that midwives expressed ambiguity regarding augmentation with oxytocin. They asserted that despite the known potency of the drug, there was an adamant belief in its efficacy, and it was at times used unnecessarily^31^. While this study cannot draw any conclusions regarding the optimal use of oxytocin for augmenting spontaneous labour or identify over- or underuse at specific obstetric units, the persistent or exacerbated variations, even after adjusting for risk factors and conducting interaction analyses, suggest unjustified differences in the use of oxytocin augmentation across the country. Creating national recommendations might be one way to enhance the standardisation of practices.

### Strengths and limitations

The strengths of this study include population-based prospectively collected data. The Swedish Pregnancy Register offers substantial opportunities for investigating pregnancy and childbirth using validated, high-quality data, including oxytocin for augmentation and annual birth rates for all obstetric units^17^.

In addition to the strengths of the study, some limitations require consideration. Due to incomplete data transfer on oxytocin from some obstetric units to the register, four units were excluded from the cohort. As a result, the study covers 40 out of 44 obstetric units representing 17 of 19 regions in Sweden. The excluded units are distributed across various parts of the country, and the regions not included (Värmland and Västmanland) only comprise one obstetric unit each. Thus, the exclusion of these units from our cohort is not expected to significantly impact the results or the generalizability of the findings.

Another limitation that needs to be acknowledged is that the Swedish Pregnancy Register does not contain information on either the time point for starting oxytocin or the duration of labour. Previous research has found that the use of oxytocin augmentation is associated with labour duration^2,3,34^. Not being able to consider the impact of this variable may thus have resulted in residual confounding. Additionally, we did not have information on underlying diseases or pregnancy-related complications such as gestational diabetes and pre-eclampsia. Since these conditions often lead to women being induced^35^, the number of women with high-risk pregnancies in our cohort is likely limited. Furthermore, previous research investigating variations in the use of other clinical interventions and where case-mix were adjusted for, did not alter their results significantly^23,24^.

The subgroup analysis which aimed to investigate the potential impact of epidural on the association between use of oxytocin and obstetric unit resulted in a relatively small number of observations for certain units. Consequently, findings pertaining to these specific obstetric units should be interpreted with caution.

## Conclusion

The variations in use of oxytocin for augmenting spontaneous labour among women in Robson groups 1 and 3 remained despite adjusting for risk factors associated with the use of oxytocin. The largest variations were found among women in Robson group 3, suggesting unjustified differences in the use of oxytocin in clinical practice. Establishing national recommendations could improve the standardising of practices. Future research is needed to investigate whether variations in the use of oxytocin impact maternal and infant outcomes and to further explore the non-clinical factors associated with its use.

## Supplementary Information

Supplementary Information.

## Data Availability

The data supporting the findings of this study is stored in the Unit of Clinical Epidemiology at Karolinska Institutet, Stockholm, Sweden. Public sharing of this data is not permitted. However, the data is available from the corresponding author on reasonable request and any researcher can access the data by obtaining ethical approval from a regional ethical review board.
