# Supplementary material for: Variations in the use of oxytocin for augmentation of labour in Sweden: a population-based cohort study
Source: Sci Rep. 2024 Jul 30;14:17483. doi: 10.1038/s41598-024-68517-1 (PMC11289380; doi:10.1038/s41598-024-68517-1)
Supplement: Supplementary file 1 — Supplementary Information. [file 41598_2024_68517_MOESM1_ESM.docx]

Table S1. Crude and adjusted risk ratios and 95% confidence intervals for oxytocin augmentation during labour by maternal and infant characteristics and hospital annual birth rate among 241 608 women stratified by Robson groups 1 and 3, Sweden, 2018-2021

|  | Oxytocin augmentation | | | |
| --- | --- | --- | --- | --- |
|  | N of women = 241 608 | | | |
|  | **Robson group 1** (n=106 676) | | **Robson group 3** (n=134 932) | |
|  | Crude risk ratio  (95% CI) | Adjusted risk ratio*  (95% CI) | Crude risk ratio  (95% CI) | Adjusted risk ratio*  (95% CI) |
| Maternal characteristics |  |  |  |  |
| Age (years) |  |  |  |  |
| <25 | 0.86 (0.84–0.87) | 0.83 (0.82–0.85) | 0.98 (0.93-1.04) | 0.93 (0.87–1.00) |
| 25-29 | 1.00 (ref) | 1.00 (ref) | 1.00 (ref) | 1.00 (ref) |
| 30-34 | 1.07 (1.05-1.08) | 1.08 (1.06-1.09) | 1.0 (0.97-1.03) | 1.04 (1.00-1.08) |
| ≥35 | 1.12 (1.11-1.14) | 1.15 (1.13-1.17) | 1.16 (1.12-1.20) | 1.22 (1.17-1.27) |
|  |  |  |  |  |
| BMI (kg/m²) |  |  |  |  |
| Underweight (<18.5) | 0.91 (0.88–0.94) | 0.98 (0.95-1.01) | 0.83 (0.76–0.92) | 0.94 (0.85-1.04) |
| Normal weight (18.5-24.9) | 1.00 (ref) | 1.00 (ref) | 1.00 (ref) | 1.00 (ref) |
| Overweight (25-29.9) | 1.05 (1.03-1.07) | 1.01 (1.0-1.02) | 1.15 (1.11-1.18) | 1.05 (1.02-1.09) |
| Obese (≥30) | 1.05 (1.03-1.07) | 1.02 (1.01-1.04) | 1.32 (1.28-1.37) | 1.14 (1.09-1.18) |
|  |  |  |  |  |
| Height (cm) |  |  |  |  |
| <155 | 1.15 (1.12-1.18) | 1.24 (1.21-1.27) | 1.65 (1.57-1.74) | 1.60 (1.50-1.72) |
| 155-164 | 1.06 (1.05-1.07) | 1.11 (1.10-1.12) | 1.24 (1.21-1.28) | 1.27 (1.23-1.31) |
| 165-174 | 1.00 (ref) | 1.00 (ref) | 1.00 (ref) | 1.00 (ref) |
| ≥175 | 0.93 (0.91-0.94) | 0.88 (0.86-0.90) | 0.87 (0.83-0.92) | 0.85 (0.81-0.90) |
|  |  |  |  |  |
| Education level |  |  |  |  |
| No formal schooling | 0.91 (0.85-0.97) | 0.93 (0.88-0.99) | 1.19 (1.11-1.28) | 1.01 (0.93-1.09) |
| Primary | 0.94 (0.92-0.97) | 0.98 (0.95-1.00) | 1.12 (1.06-1.18) | 1.03 (0.98-1.10) |
| Secondary | 1.00 (ref) | 1.00 (ref) | 1.00 (ref) | 1.00 (ref) |
| Post-secondary | 1.04 (1.03-1.06) | 0.99 (0.98-1.00) | 0.87 (0.84-0.89) | 0.91 (0.88-0.94) |
|  |  |  |  |  |
| Country of birth |  |  |  |  |
| Nordic | 1.00 (ref) | 1.00 (ref) | 1.00 (ref) | 1.00 (ref) |
| Asia | 1.11 (1.08-1.14) | 1.08 (1.04-1.11) | 1.24 (1.17-1.33) | 1.17 (1.08-1.26) |
| Africa | 0.98 (0.95-1.01) | 1.03 (1.00-1.06) | 1.40 (1.34-1.46) | 1.23 (1.16-1.30) |
| South America | 1.17 (1.12-1.23) | 1.12 (1.05-1.19) | 1.53 (1.35-1.73) | 1.41 (1.23-1.62) |
| Other | 1.04 (1.03-1.06) | 1.06 (1.04-1.07) | 1.16 (1.12-1.20) | 1.10 (1.06-1.15) |
|  |  |  |  |  |
| Epidural analgesia |  |  |  |  |
| Yes | 2.31 (2.28-2.35) | 2.18 (2.15-2.22) | 3.28 (3.21-3.36) | 3.31 (3.22-3.40) |
|  |  |  |  |  |
| Infant characteristics |  |  |  |  |
| Birthweight (g) |  |  |  |  |
| <3000 | 0.83 (0.81-0.84) | 0.83 (0.82-0.85) | 0.90 (0.85-0.95) | 0.86 (0.80-0.92) |
| 3000-3499 | 1.00 (ref) | 1.00 (ref) | 1.00 (ref) | 1.00 (ref) |
| 3500-3999 | 1.14 (1.13-1.15) | 1.13 (1.11-1.14) | 1.21 (1.18-1.25) | 1.22 (1.18-1.27) |
| ≥4000 | 1.28 (1.27-1.30) | 1.27 (1.25-1.29) | 1.60 (1.54-1.65) | 1.60 (1.54-1.66) |
|  |  |  |  |  |
| Gestational week |  |  |  |  |
| 37+0 - 38+6 | 1.00 (ref) | 1.00 (ref) | 1.00 (ref) | 1.00 (ref) |
| 39+0 - 40+6 | 1.22 (1.20-1.24) | 1.12 (1.10-1.14) | 1.14 (1.10-1.18) | 1.03 (0.99-1.08) |
| 41+0 - 41+6 | 1.44 (1.41-1.46) | 1.27 (1.24-1.29) | 1.50 (1.44-1.57) | 1.26 (1.20-1.33) |
| ≥42 | 1.60 (1.55-1.65) | 1.42 (1.37-1.47) | 2.31 (2.11-2.53) | 1.86 (1.68-2.07) |
|  |  |  |  |  |
| Hospital annual birth rate |  |  |  |  |
| <1000 | 1.04 (1.02-1.06) | 1.05 (1.03-1.07) | 1.13 (1.08-1.18) | 1.12 (1.07-1.18) |
| 1000-2499 | 1.00 (ref) | 1.00 (ref) | 1.00 (ref) | 1.00 (ref) |
| 2500-3999 | 1.02 (1.01-1.03) | 1.0 (0.99-1.01) | 0.98 (0.95-1.01) | 0.97 (0.94-1.00) |
| ≥4000 | 0.98 (0.97-0.99) | 0.96 (0.94-0.97) | 0.78 (0.76-0.81) | 0.78 (0.75-0.81) |

*Adjusted for maternal age, BMI, height, education level, country of birth, infant birthweight, gestational week, and hospital annual birth rate

Table S2. Crude and adjusted risk differences and 95% confidence intervals for oxytocin augmentation during labour by obstetric unit among 106 676 women in Robson group 1, Sweden, 2018- 2021

|  | Oxytocin augmentation during labour | | | |
| --- | --- | --- | --- | --- |
|  | **Robson group 1** | | | |
|  | N total | n eventᵃ (%) | Crude risk difference  (95% CI) | Adjusted risk differenceᵇ  (95% CI) |
| Obstetric unit |  |  |  |  |
| Sweden | 106676 | 63283 (59.3) | Ref (1.00) | Ref (1.00) |
| Ystad | 1588 | 738 (46.5) | -13.04 (-15.51 to -10.57) | -14.39 (-17.10 to -11.68) |
| Göteborg | 10960 | 5475 (50.0) | -10.44 (-11.43 to -9.45) | -11.78 (-13.12 to -10.43) |
| Kristianstad | 1814 | 876 (48.3) | -11.22 (-13.54 to -8.90) | -11.05 (-13.61 to -8.48) |
| Visby | 500 | 267 (53.4) | -5.95 (-10.33 to -1.57) | -10.69 (-15.70 to -5.69) |
| Örebro | 3351 | 1667 (49.8) | -9.89 (-11.61 to -8.17) | -10.47 (-12.32 to -8.62) |
| Värnamo | 1039 | 594 (57.2) | -2.17 (-5.20 to 0.85) | -7.35 (-10.94 to -3.77) |
| Varberg | 2397 | 1302 (54.3) | -5.12 (-7.14 to -3.10) | -7.33 (-10.08 to -4.58) |
| Eksjö | 1264 | 668 (52.9) | -6.55 (-9.32 to -3.78) | -5.20 (-8.18 to -2.22) |
| Trollhättan | 3482 | 1936 (55.6) | -3.85 (-5.53 to -2.17) | -4.50 (-6.40 to -2.60) |
| Halmstad | 1866 | 980 (52.5) | -6.93 (-9.21 to -4.64) | -4.14 (-6.92 to -1.36) |
| Karlskrona | 1678 | 945 (56.3) | -3.05 (-5.45 to -0.66) | -3.52 (-6.15 to -0.89) |
| Sundsvall | 1616 | 908 (56.2) | -3.18 (-5.62 to -0.75) | -3.16 (-5.79 to -0.53) |
| Huddinge Karolinska | 4431 | 2587 (58.4) | -0.98 (-2.46 to 0.50) | -2.85 (-4.53 to -1.18) |
| Södertälje | 3151 | 1845 (58.6) | -0.79 (-2.54 to 0.95) | -2.80 (-4.82 to -0.78) |
| BB Stockholm | 4908 | 2984 (60.8) | 1.56 (0.15 to 2.95) | -1.55 (-3.14 to 0.04) |
| Falun | 2917 | 1636 (56.1) | -3.33 (-5.15 to -1.50) | -1.06 (-3.07 to 0.94) |
| Jönköping | 2249 | 1347 (59.9) | 0.58 (-1.46 to 2.63) | -0.52 (-2.77 to 1.73) |
| Örnsköldsvik | 549 | 335 (61.0) | 1.71 (-2.38 to 5.80) | -0.22 (-4.76 to 4.33) |
| Helsingborg | 3429 | 2043 (59.6) | 0.27 (-1.40 to 1.94) | 0.06 (-1.88 to 2.00) |
| Skövde | 2818 | 1684 (59.8) | 0.45 (-1.39 to 2.28) | 0.48 (-1.56 to 2.52) |
| Borås | 3208 | 1961 (61.1) | 1.86 (0.15 to 3.57) | 0.58 ('-1.37 to 2.53) |
| Lycksele | 336 | 207 (61.6) | 2.29 (-2.92 to 7.50) | 1.39 (-4.71 to 7.50) |
| Nyköping | 1032 | 639 (61.9) | 2.62 (-0.36 to 5.60) | 1.40 (-2.30 to 5.09) |
| Malmö | 5255 | 3086 (58.7) | -0.63 (-1.99 to 0.74) | 1.65 (0.07 to 3.24) |
| Västervik | 926 | 580 (62.6) | 3.34 (0.21 to 6.47) | 2.17 (-1.39 to 5.74) |
| Skellefteå | 828 | 531 (64.1) | 4.85 (1.57 to 8.13) | 2.61 (-1.19 to 6.40) |
| Sunderby | 1237 | 788 (63.7) | 4.43 (1.74 to 7.13) | 3.18 (0.33 to 6.07) |
| Gällivare | 341 | 227 (66.6) | 7.27 (2.25 to 12.28) | 3.29 (-2.18 to 8.77) |
| Kalmar | 1817 | 1117 (61.5) | 2.19 (-0.07 to 4.45) | 3.46 (1.12 to 5.80) |
| Danderyd | 6952 | 4320 (62.1) | 3.01 (1.83 to 4.19) | 3.89 (2.51 to 5.27) |
| Eskilstuna | 1846 | 1165 (63.1) | 3.85 (1.63 to 6.07) | 3.97 (1.56 to 6.37) |
| Östersund | 1291 | 820 (63.5) | 4.25 (1.60 to 6.89) | 4.08 (1.23 to 6.94) |
| Gävle | 1643 | 1041 (63.4) | 4.10 (1.75 to 6.45) | 5.14 (2.66 to 7.63) |
| Solna Karolinska | 3373 | 2230 (66.1) | 7.01 (5.39 to 8.64) | 5.22 (3.36 to 7.09) |
| Lund | 3268 | 2117 (64.8) | 5.63 (3.96 to 7.29) | 5.39 (3.53 to 7.25) |
| Hudiksvall | 983 | 649 (66.0) | 6.76 (3.80 to 9.74) | 5.56 (2.15 to 8.96) |
| Södersjukhuset | 8707 | 5607 (64.4) | 5.52 (4.47 to 6.58) | 6.98 (5.74 to 8.21) |
| Umeå | 1971 | 1329 (67.4) | 8.26 (6.17 to 10.35) | 8.47 (6.22 to 10.72) |
| Linköping | 3171 | 2238 (70.6) | 11.60 (9.99 to 13.21) | 11.68 (9.94 to 13.42) |
| Norrköping | 2484 | 1814 (73.0) | 14.03 (12.26 to 15.80) | 13.89 (12.00 to 15.78) |

*Note:* Crude and adjusted risk differences contrasted the risk of oxytocin augmentation at each obstetric unit versus the rest of Sweden

ᵃ Number of women with oxytocin augmentation during labour

ᵇ Adjusted for maternal age, BMI, height, education level, country of birth, infant birthweight, gestational week, and hospital annual birth rate

Table S3. Crude and adjusted risk differences and 95% confidence intervals for oxytocin augmentation during labour by obstetric unit among 134 932 women in Robson group 3, Sweden, 2018-2021

|  | Oxytocin augmentation | | | |
| --- | --- | --- | --- | --- |
|  | **Robson group 3** | | | |
|  | n total | n eventᵃ (%) | Crude risk difference  (95% CI) | Adjusted risk differenceᵇ  (95% CI) |
| Obstetric unit |  |  |  |  |
| Sweden | 134932 | 21213 (15.7) | Ref (1.00) | Ref (1.00) |
| Jönköping | 2947 | 304 (10.3) | -5.53 (-6.64 to -4.41) | -6.74 (-7.86 to -5.61) |
| Ystad | 1823 | 191 (10.5) | -5.32 ('-6.74 to -3.90) | -6.46 (-7.92 to -4.99) |
| Kalmar | 2366 | 241 (10.2) | -5.63 (-6.87 to -4.40) | -6.10 (-7.35 to -4.85) |
| Halmstad | 2610 | 266 (10.2) | -5.64 (-6.82 to -4.46) | -5.52 (-6.94 to -4.10) |
| Kristianstad | 2864 | 333 (11.6) | -4.18 (-5.37 to -2.99) | -5.05 (-6.32 to -3.78) |
| Örebro | 4272 | 504 (11.8) | -4.05 (-5.04 to -3.06) | -5.01 (-6.01 to -4.01) |
| Eksjö | 1899 | 232 (12.2) | -3.55 (-5.04 to -2.07) | -4.78 (-6.27 to -3.30) |
| Visby | 623 | 74 (11.9) | -3.86 (-6.41 to -1.31) | -4.81 (-7.39 to -2.24) |
| Västervik | 1236 | 179 (14.5) | -1.25 (-3.22 to 0.72) | -4.32 (-6.15 to -2.35) |
| Huddinge Karolinska | 5629 | 726 (12.9) | -2.95 (-3.84 to -2.05) | -4.13 (-5.07 to -3.19) |
| Göteborg | 12229 | 1333 (10.9) | -5.30 (-5.89 to -4.71) | -3.62 (-4.50 to -2.74) |
| Södertälje | 3246 | 417 (12.9) | -2.95 (-4.11 to -1.78) | -3.51 (-4.78 to -2.25) |
| BB Stockholm | 4860 | 607 (12.5) | -3.35 (-4.30 to -2.40) | -3.35 (-4.41 to -2.28) |
| Sundsvall | 2205 | 318 (14.4) | -1.32 (-2.80 to 0.16) | -3.05 (-4.53 to -1.56) |
| Varberg | 2715 | 367 (13.5) | -2.25 (-3.55 to -0.95) | -2.90 (-4.48 to -1.32) |
| Helsingborg | 4844 | 662 (13.7) | -2.13 (-3.12 to -1.14) | -2.60 (-3.74 to -1.47) |
| Lycksele | 527 | 87 (16.5) | 0.79 (-2.39 to 3.97) | -2.71 (-5.94 to 0.52) |
| Örnsköldsvik | 921 | 152 (16.5) | 0.79 (-1.62 to 3.19) | -2.56 (-4.82 to -0.30) |
| Karlskrona | 2309 | 333 (14.4) | -1.32 (-2.77 to 0.12) | -2.16 (-3.74 to -0.57) |
| Falun | 4075 | 603 (14.8) | -0.94 (-2.06 to 0.16) | -1.71 (-2.93 to -0.50) |
| Solna Karolinska | 3570 | 572 (16.0) | 0.31 (-0.91 to 1.53) | -1.58 (-2.86 to -0.30) |
| Värnamo | 1542 | 263 (17.1) | 1.35 (-0.54 to 3.24) | -1.62 (-3.49 to 0.43) |
| Hudiksvalls | 1511 | 287 (19.0) | 3.31 (1.32 to 5.30) | -0.41 (-2.36 to -1.54) |
| Danderyd | 8420 | 1118 (13.3) | -2.61 (-3.36 to -1.85) | -0.11 (-1.17 to 0.94) |
| Lund | 4442 | 700 (15.8) | 0.04 (-1.05 to 1.13) | -0.10 (-1.33 to 1.12) |
| Skellefteå | 1158 | 218 (18.8) | 3.13 (0.87 to 5.39) | -0.14 (-2.42 to 2.14) |
| Gävle | 2244 | 395 (17.6) | 1.91 (0.33 to 3.50) | 0.19 (-1.45 to 1.83) |
| Trollhättan | 4848 | 835 (17.2) | 1.56 (0.48 to 2.64) | 0.60 (-0.62 to 1.82) |
| Umeå | 2323 | 411 (17.7) | 2.01 (0.44 to 3.57) | 0.63 (-1.01 to 2.27) |
| Skövde | 3644 | 690 (18.9) | 3.30 (2.02 to 4.59) | 1.23 (-0.15 to 2.61) |
| Malmö | 6627 | 954 (14.4) | -1.39 (-2.26 to -0.53) | 1.45 (0.22 to 2.67) |
| Borås | 4107 | 732 (17.8) | 2.17 (0.98 to 3.35) | 2.85 (1.43 to 4.27) |
| Södersjukhuset | 8892 | 1306 (14.7) | -1.11 (-1.87 to -0.34) | 3.11 (1.98 to 4.24) |
| Nyköping | 1360 | 311 (22.9) | 7.22 (4.98 to 9.46) | 4.40 (1.98 to 6.83) |
| Östersund | 1649 | 387 (23.5) | 7.84 (5.79 to 9.90) | 7.14 (4.93 to 9.35) |
| Eskilstuna | 2889 | 746 (25.8) | 10.32 (8.71 to 11.93) | 8.18 (6.49 to 9.88) |
| Norrköping | 3290 | 841 (25.6) | 10.09 (8.58 to 11.59) | 9.43 (7.80 to 11.05) |
| Gällivare | 877 | 260 (29.7) | 14.02 (10.99 to 17.05) | 11.15 (8.00 to 14.30) |
| Sunderby | 3208 | 888 (27.7) | 12.25 (10.69 to 13.81) | 12.13 (10.40 to 13.85) |
| Linköping | 4131 | 1370 (33.2) | 17.99 (16.54 to 19.44) | 19.25 (17.55 to 20.95) |

*Note:* Crude and adjusted risk differences contrasted the risk of oxytocin augmentation at each obstetric unit versus the rest of Sweden

ᵃ Number of women with oxytocin augmentation during labour

ᵇ Adjusted for maternal age, BMI, height, education level, country of birth, infant birthweight, gestational week, and hospital annual birth rate

Table S4. Interaction effect between obstetric unit and epidural analgesia on oxytocin augmentation during labour for 241 608 women in Robson groups 1 and 3, Sweden, 2018-2021

|  | Robson group 1 | Robson group 3 |
| --- | --- | --- |
| Obstetric unit | *p* for interaction* | *p* for interaction* |
| BB Stockholm | <0.001 | 0.002 |
| Borås | 0.062 | 0.048 |
| Danderyd | <0.001 | 0.001 |
| Eksjö | <0.001 | 0.112 |
| Eskilstuna | <0.001 | <0.001 |
| Falun | 0.572 | 0.113 |
| Gällivare | 0.041 | 0.001 |
| Gävle | 0.678 | 0.003 |
| Göteborg | 0.494 | <0.001 |
| Halmstad | 0.456 | <0.001 |
| Helsingborg | 0.578 | 0.384 |
| Huddinge Karolinska | 0.036 | 0.003 |
| Hudiksvall | 0.220 | 0.921 |
| Jönköping | 0.139 | <0.001 |
| Kalmar | 0.607 | 0.088 |
| Karlskrona | 0.692 | 0.001 |
| Kristianstad | 0.160 | 0.194 |
| Linköping | <0.001 | <0.001 |
| Lund | 0.982 | 0.199 |
| Lycksele | 0.954 | 0.649 |
| Malmö | 0.025 | 0.001 |
| Norrköping | <0.001 | <0.001 |
| Nyköping | 0.448 | <0.001 |
| Skellefteå | 0.070 | 0.824 |
| Skövde | 0.628 | 0.122 |
| Solna Karolinska | <0.001 | 0.001 |
| Södersjukhuset | <0.001 | 0.036 |
| Sunderby | 0.258 | 0.017 |
| Sundsvall | <0.001 | 0.366 |
| Södertälje | 0.152 | 0.141 |
| Trollhättan | 0.001 | <0.001 |
| Umeå | 0.528 | 0.002 |
| Varberg | 0.401 | 0.162 |
| Visby | 0.274 | 0.673 |
| Värnamo | <0.001 | 0.220 |
| Västervik | 0.101 | 0.300 |
| Ystad | 0.962 | 0.037 |
| Örebro | <0.001 | 0.055 |
| Örnsköldsvik | 0.102 | 0.311 |
| Östersund | 0.144 | <0.001 |

*Adjusted for maternal age, BMI, height, education level, country of birth, infant birthweight, gestational week and hospital annual birth rate

Table S5. Characteristics of the 40 obstetric units included in the study

| Obstetric unit | Funding | Teaching status | Annual birth rate* | NICU |
| --- | --- | --- | --- | --- |
| Göteborg | Public | University teaching hospital | 8700 | Yes |
| Södersjukhuset | Public | General hospital | 6400 | Yes |
| Danderyd | Public | General hospital | 5500 | Yes |
| Malmö | Public | University teaching hospital | 4500 | Yes |
| Karolinska Huddinge | Public | University teaching hospital | 3800 | Yes |
| BB Stockholm | 49% public, 51% private | General hospital | 3500 | Yes |
| Lund | Public | University teaching hospital | 3000 | Yes |
| Helsingborg | Public | General hospital | 3000 | Yes |
| Trollhättan | Public | General hospital | 2900 | Yes |
| Örebro | Public | University teaching hospital | 2800 | Yes |
| Karolinska Solna | Public | University teaching hospital | 2800 | Yes |
| Borås | Public | General hospital | 2700 | Yes |
| Falun | Public | General hospital | 2700 | Yes |
| Linköping | Public | University teaching hospital | 2500 | Yes |
| Skövde | Public | General hospital | 2300 | Yes |
| Södertälje | Public | General hospital | 2100 | No |
| Varberg | Public | General hospital | 2000 | No |
| Norrköping | Public | General hospital | 1900 | Yes |
| Jönköping | Public | General hospital | 1800 | Yes |
| Kristianstad | Public | General hospital | 1800 | Yes |
| Halmstad | Public | General hospital | 1800 | Yes |
| Eskilstuna | Public | General hospital | 1700 | Yes |
| Sunderby | Public | General hospital | 1700 | Yes |
| Umeå | Public | University teaching hospital | 1500 | Yes |
| Gävle | Public | General hospital | 1500 | Yes |
| Sundsvall | Public | General hospital | 1500 | Yes |
| Kalmar | Public | General hospital | 1400 | Yes |
| Karlskrona | Public | General hospital | 1300 | Yes |
| Östersund | Public | General hospital | 1200 | Yes |
| Ystad | Public | General hospital | 1200 | No |
| Eksjö | Public | General hospital | 1100 | No |
| Hudiksvall | Public | General hospital | 900 | Yes |
| Värnamo | Public | General hospital | 900 | No |
| Nyköping | Public | General hospital | 900 | Yes |
| Västervik | Public | General hospital | 800 | Yes |
| Skellefteå | Public | General hospital | 700 | Yes |
| Örnsköldsvik | Public | General hospital | 600 | Yes |
| Gällivare | Public | General hospital | 400 | Yes |
| Visby | Public | General hospital | 400 | Yes |
| Lycksele | Public | General hospital | 300 | No |

NICU = Neonatal Intensive Care Unit

*Mean annual birth rate 2018-2021

**Figure 1.** **Flowchart of the study population**

Uncertain reporting of

data on oxytocin

n = 14 532

(Västerås Västmanlands hospital,

Karlstad Central hospital, Sollefteå hospital, Karlskoga hospital)

Robson groups

2, 4, 5A, 5B, 5C, 6-10

n = 116 942

IUFD

(Intra-uterine fetal demice)

n = 183

Study cohort

241 608 births in Robson group 1* and 3**

Robson group 1

n = 106 676

Robson group 3

n = 134 932

Dataset from the Swedish Pregnancy Register

373 265 births, 2018-2021

*Nulliparous, singleton, cephalic, term (≥37+0), spontaneous onset of labour

**Multiparous, no previous caesarean section, singleton, cephalic, term (≥37+0), spontaneous onset of labour
